# Uncovering Spatiotemporal Characteristics of Human Online Behaviors during Extreme Events

**DOI:** 10.1371/journal.pone.0138673

**Published:** 2015-10-22

**Authors:** Chao Gao, Jiming Liu

**Affiliations:** 1 College of Computer and Information Science, Southwest University, Chongqing, China; 2 Department of Computer Science, Hong Kong Baptist University, Kowloon Tong, Hong Kong; 3 Key Laboratory of Symbolic Computation and Knowledge Engineering of Ministry of Education, Jilin University, Changchun, China; Hangzhou Normal University, CHINA

## Abstract

In response to an extreme event, individuals on social media demonstrate interesting behaviors depending on their backgrounds. By making use of the large-scale datasets of posts and search queries collected from Twitter and GoogleTrends, we first identify the distinct categories of human collective online concerns and durations based on the distributions of solo tweets and new incremental tweets about events. Such a characterization enables us to gain a better understanding of dynamic changes in human behaviors corresponding to different types of events. Next, we observe the heterogeneity of individual responses to events through measuring the fraction of event-related tweets relative to the tweets released by an individual, and thus empirically confirm the heterogeneity assumption as adopted in the meta-population models for characterizing collective responses to events. Finally, based on the correlations of information entropy in different regions, we show that the observed distinct responses may be caused by their different speeds in information propagation. In addition, based on the detrended fluctuation analysis, we find that there exists a self-similar evolution process for the collective responses within a region. These findings have provided a detailed account for the nature of distinct human behaviors on social media in presence of extreme events.

## Introduction

Understanding the characteristics and dynamic changes of collective online behaviors during an extreme event is a very important issue for emergency management [1, 2]. Specifically, due to the uncertainty of an extreme event [3], exploring heterogeneous characteristics of human online behaviors can enhance emergency situation awareness for disaster relief (e.g., monitoring collective panic behaviors). And a clear classification of human behaviors can help the emergency management to effectively deal with the event based on the past experience. For example, the emergency management can gain a better understanding of the dynamic human behaviors in different regions during an ongoing event and take effective measures (e.g., humanitarian relief or information management) in advance [4].

Previous studies have shown that human behaviors, during an extreme event, can be affected by various factors, e.g., the specific profiles of people (such as geographical/regional profiles) [5] and social influences (e.g., influences from friends and public news) [6]. As a result, different kinds of human responses may emerge or disappear on a daily basis. An interesting question that one may raise is: Is the evolution of human collective online behaviors over time mostly random, or governed by certain intrinsic rules? At the moment, it presents a challenge to adequately address such a question due to the lack of appropriate means for characterizing human behaviors and for quantitatively measuring their changes over time.

The existing questionnaire-based studies have provided a qualitative method for analyzing human collective behaviors to a certain extent [7]. However, they cannot reveal the dynamic changes of such behaviors over time. Meanwhile, the conclusions of a questionnaire-based analysis are often drawn from limited surveillance data, which may not always be evidentially convincing enough. As a rapidly emerging communication channel, online media can readily be used to study and hence explain the basic individual behaviors (e.g., to detect collective sentiments [8, 9], dynamics of human interests [10] and mobility [11]) and their hidden interactions (e.g., the structural detection based on retweets) [12, 13], which have been applied to improve disaster relief (e.g., earthquake [14], H1N1 [15, 16]) and to track a financial crisis [17].

In essence, online social media have provided an important data source for probing into the dynamics of human collective behaviors, while uncovering various patterns and phenomena as well as possible scaling laws [18, 19]. Along this direction, some human collective online behaviors during events have been examined with respect to their temporal scale, such as the features of sudden increment and power-law decay in the time series data composed of tweets count or search queries changing over time [18, 20], which have further been used to reveal the type of an event caused by exogenous or endogenous factors [21]. However, they overestimate the effect of the highest peak (measured by the highest volume in a normalized time series, as shown in Fig A in S1 Appendix) and assume each individual has the same contribution to the time series data. In reality, an event can consist of a series of episodes (i.e., subevents) [3, 22], which may cause multiple peaks (e.g., the double peak phenomenon in Fig A(a2) in S1 Appendix) in the time series. Although multiple peaks have been considered in [23, 24] when distinguishing collective attentions based on the sliding windows and peak-detection techniques, the homogenous assumption in their studies is somewhat controversial, as it does not take into account the differences between individual behaviors and thus assumes people have the same interests in events. On the contrary, empirical studies on Twitter have found that some people carry out a certain behavior during an event repeatedly (i.e., more event-related tweets are released by an account). That is to say, people with different profiles (e.g., regional information) will have varying contributions to the collective information-sharing behavior (i.e., tweet count). In this regard, the conclusion drawn from the homogenous assumption neither addresses the distinct contribution of each individual if only counting the total number of tweets, nor assesses the contribution of individual behaviors to overall influences if only measuring the total number of people.

In this paper, we describe a data analytics method to reveal heterogenous characteristics of collective human responses to events from the individual perspective and demonstrate it by using the time series data from two online sources, i.e., tweets and search queries about events from Twitter and GoogleTrends, respectively. Specifically, we define and compute two temporal-scale indicators for measuring the characteristics of these time series (i.e., the cumulative distribution of solo tweets about events that reflects the diversity of individual concerns about events, and the distribution of increment of tweets about events which reflects the durations of events), so as to better identify different types of events. In addition, it also performs spatial-scale analysis, which is to uncover regional characteristics of information propagation through measuring the correlation of regional information entropy, and to reveal the regional self-similarity evolution characteristics of human collective online responses based on the detrended fluctuation analysis. The distinct feature of this work lies in its focus on the heterogeneous characteristics of individual behaviors [25] that can result in different contributions to the collective behavior measurements (e.g., the dynamic changes of time series of tweets), in terms of the spatiotemporal patterns of different public concerns [15] (or interests [26]).

## Materials and Methods

### Data collection

**Fig 1:**
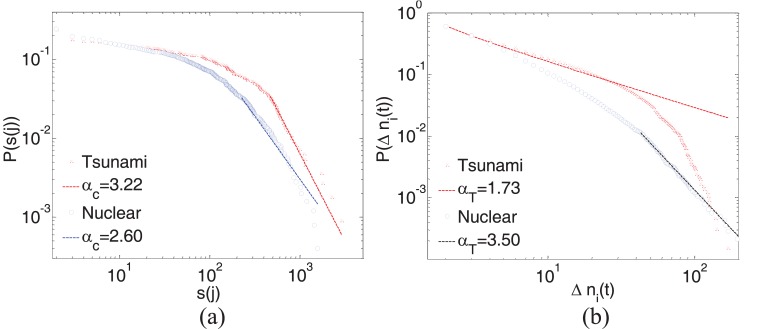
Distributions of collective online behaviors. (a) the cumulative distribution of solo tweets about two hashtags and their exponents *α*_*c*_, and (b) the corresponding distribution of incremental tweets about two hashtags and their exponents *α*_*t*_. All exponents are estimated by applying KS statistical test.

**Fig 2:**
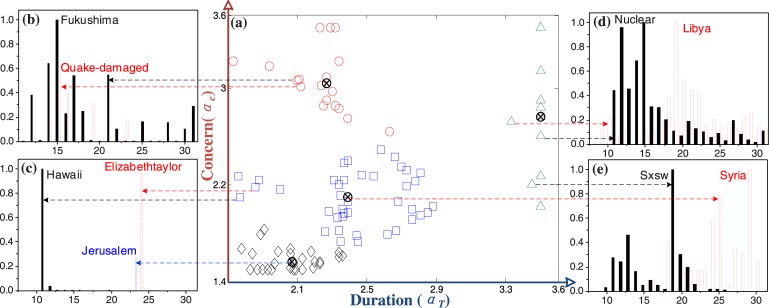
The illustration of classification in the *CD* space based on the ranking values of human concerns and durations (*k* = 4 in KM algorithm). Some events, as shown in (c) and (e), are categorized into groups even if they have similar shapes of time series. Through comparing the temporal dynamic of collective online behaviors from Twitter, we find that our method can overcome the effect of multiple peaks in the curve, and provide more precise classification even if two events have similar shape of time series data.

By snowball sampling using Twitter REST API [27], we first extracted publicly available Twitter data from March 8 to March 31, 2011, as shown in Table A in S1 Appendix. In this period, there existed many events that can be used to differentiate collective online behaviors in presence of social unrests (e.g., Libya crisis) and extreme events (e.g., earthquake, tsunami or nuclear crisis). Specifically, these events can be described as instantaneous and non-instantaneous events based on the growth trends of public news about events [28]. Due to the infrequency and potential damages of extreme events to human beings (i.e., the relative infrequency of occurrence and unpredictable subsequent consequences), people will adapt proper behaviors to reduce the uncertainty and potential threat according to the types of events and perceived risks. On the one hand, for a familiar event (e.g., tsunami or the death of Elizabeth Taylor), since people are aware of their potential impacts and/or damages, they will quickly send messages via social media to warn others and/or cherish the memory of a famous actor. After that, people’s attention will be attracted to other topics. Meanwhile, although most of the people take part in such discussions, the messages they sent are just re-tweets from others. On the other hand, for some uncertain events, especially for their unpredictable subsequent consequences (e.g., nuclear leaking or Lybia crisis), people share information via social media and take part in event-related discussions for gaining more information and reduce their uncertainties. During such events, we can extract more solo tweets (which mean more different opinions related to an event are emerged) and find that such discussions will last for a relatively long time. Therefore, this paper first selects two typical events as shown in Fig 1 to illustrate the different characteristics of events, and then classifies events from the individual perspective through measuring different responses to events as shown in Fig 2.

After that, we extracted the corresponding event data from GoogleTrends based on event-related keywords. Based on the GoogleTrends data, we can estimate the effect of solo tweets on event classification (as shown in Fig E in S1 Appendix). Although the Twitter data may not reflect all characteristics of our society due to the intrinsic pitfalls of social media [29] that can cause some biases (e.g., the analysis result of regional activeness in Fig H in S1 Appendix), the conclusion based on Twitter can reflect the characteristics of human online behaviors, which will in turn reveal human behaviors in the real world to a certain extent.

### Hashtag selection based on the semantic analysis

In social media, hashtag is a popular technique to represent an event or object in tweets [15, 19, 24]. However, such keyword-based analysis cannot address the effects of different event episodes on collective responses [30]. Moreover, some irrelevant contents and typos in tweets may affect the quality of results. Here, a clustering-based analysis method [30] is used to eliminate the noises of irrelevant contents. First, based on the co-occurrence of hashtags appearing in a tweet, a weighted hashtag network can be constructed in which a node denotes a hashtag and each edge is associated with a normalized weight. Based on such network, the skeleton of information contents on social media is plotted in Fig B in S1 Appendix. From this figure, we find that each event contains a few core hashtags and many marginal hashtags, which is a typical core-periphery structure. Then, based on the modularity *Q* of a network defined in [31], we can categorize hashtags into different groups and identify the core hashtags. In this step, some core-periphery structures were found from the hashtag network as shown in Fig B(a), Fig B(b) and Fig B(c) in S1 Appendix. Finally, those core hashtags were used to represent events and searched in GoogleTrends in order to estimate the effect of solo tweets on event classification.

### Measurements

During an extreme event (or exogenous disturbance), people are often selective to the type of information that they are concerned about most [32], to which different individuals (e.g., due to their specific beliefs and/or feelings) may respond differently, resulting in different behaviors. Generally speaking, whether or not people post a tweet or seek event-related information is determined by the level of their concerns and the durations of the event. Here, we consider three measurements to reveal the heterogeneous spatiotemporal characteristics of human collective online behaviors. The first two measurements, drawn from the (i) statistical distribution of sole tweets about an event and (ii) distribution of new incremental tweets about an event, are used for measuring temporal features of human collective online behaviors. The third measurement is (iii) the level of individuals’ concern about an event, which will be used for measuring spatial features of human collective online behaviors.

(i) **Human collective concerns about events** can be measured by the statistical distribution of sole tweets. That is because the sole tweets can further represent human actives rather than retweets [19] (discussed in Sec B in S1 Appendix). The more solo tweets, the more opinions an event generates. In this paper, human collective concerns about events are measured by solo tweets about the events and their retweeted times. For a hashtag #*h*, there are a total of *m*_#*h*_ different solo tweets containing such a hashtag. And each solo tweet *j* in *m*_#*h*_ can be retweeted *s*(*j*) times.

(ii) **Durations of human collective concerns about events** can be measured by the distribution of new incremental tweets. Due to the rapid response mechanism of information spreading on Twitter, the information will quickly become outdated [33]. And people just forward and retweet the latest information. Therefore, the distribution of incremental new tweets of a hashtag (i.e., Δ*n*_*i*_(*t*)) can be used to reflect the durations of human collective concerns about events, which also further reflects the durations of the events. Here, Δ*n*_*i*_(*t*) is defined as *n*_*i*_(*t*+1) − *n*_*i*_(*t*), where *n*_*i*_(*t*) is the total number of tweets about an event on day *t*.

(iii) **Individuals’ concerns about an event** can be measured by the fraction of the number of event-related tweets that contain a certain term *i*, relative to the tweets released by a user *p*, i.e., $C_{i}^{p}=N_{i}^{p}/N^{p}$ where $C_{i}^{p}$ is the concern of a user *p* about an event *i*. $N_{i}^{p}=\sum n_{i}^{p}\left(t\right)$ is the total number of tweets about a certain term *i* released by *p*. *N* ^*p*^ = ∑*n* ^*p*^(*t*) denotes the tweets released by *p*. If a user *p* posts a tweet about an event at least once, we regard *p* as an active person. And the user *p* is sensitive to an event *i* if $C_{i}^{p}=1$. The activeness of a region for an event can be measured by the percentage of people in the region who are sensitive to the event. Based on the distribution of $C_{i}^{p}$, we can further observe and measure the heterogeneity of regional collective responses to events.

## Results

### Fat-tailed distributions of collective online behaviors and their application in event classification

Fig 1 presents two examples to reveal the unequivocal signature of the fat-tailed scaling characteristic of human collective online behaviors with distinct characteristics. More results are shown in Fig C in S1 Appendix. Given a time series of posts from Twitter (e.g., Fig A in S1 Appendix), some event-related hashtags are selected through cluster-based analysis to denote different events (Sec A in S1 Appendix). For a sole tweet *j* of a certain hashtag #*h*, the cumulative distribution *s*(*j*) follows a power-law distribution as shown in Fig 1a and their exponents (*α*_*c*_) are estimated by applying the Kolomogorov-Smirnov statistical test based on [34]. This indicates that most people are interested in only a few solo tweets, i.e., only a few solo tweets can propagate and attract most of the people. Because of the different contributions of individuals to the total number of tweets, we can observe the distinct human online responses to events (i.e., different *α*_*c*_) even if two events have similar shapes of time series. For example, although events in Fig A(a3) and Fig A(a5) in S1 Appendix have the similar dynamic changes in the tweet count over time (i.e., a single peak in the time series), they attract different concerns (i.e., different *α*_*c*_) because of the different compositions of time series (i.e., the proportion of solo tweets to all tweets as discussed in Sec B in S1 Appendix).

On the other hand, Fig 1b shows that the incremental distribution of new tweets (i.e., Δ *n*_*i*_(*t*)) also follows a power-law distribution. Although the given time series of tweets about events have the similar varying tendency, such an exponent (*α*_*t*_) can also reveal the distinct temporal characteristic of human concerns about a certain event. For example, an instantaneous event (the volume of time series data has a sudden peak and relatively rapid relaxation [21], e.g., the tsunami hit in Hawaii or the death of Elizabeth Tayor as shown in Fig A in S1 Appendix) has the fewest solo tweets, and most of the tweets in such an event just forwarded certain popular solo tweets, which to a certain extent reflects the alarming ability of social media. While a relatively non-instantaneous event (the volume of time series data has a significant growth preceding the occurrence of the event and symmetric relaxation after the event [21], e.g., Libya crisis or nuclear crisis in Japan, as shown in Fig A in S1 Appendix) has the much more solo tweets, corresponding to the diversity of opinions to the event.

Based on the distinct human responses to events, measured by the exponents of the cumulative distribution of solo tweets (i.e., *α*_*c*_) and the distribution of new incremental tweets (*α*_*t*_), an event can be plotted in a two-dimensional space with respect to (1) human *concern* about the event and (2) the *duration* of the concern (denoted as *CD* space, for short). Fig 2 takes some typical events as examples to elucidate the clustering result based on the K-means algorithm, in which each event is represented by marker symbols of different shapes whose coordinates correspond to their positions in the concern-ranking (i.e., *α*_*c*_) and duration-ranking (i.e., *α*_*t*_) values. Furthermore, Fig C in S1 Appendix plots the distribution of retweeted network and the distribution of incremental tweets about some distinct events, as shown in Fig 2b, 2c, 2d and 2e, which belong to different categories. More classification results are shown in Fig D in S1 Appendix. With these two indicators, we are capable of effectively capturing the basic characteristics of events (i.e., the attraction of events to individuals and its durations) from the perspective of individuals’ behaviors, and then categorize events into several clusters.

More specifically, previous studies on the classification of online behaviors overestimate the effect of the highest peaks in the time series due to the homogenous assumption that doesn’t distinguish the compositions in a time series data [21]. In reality, however, the time series data on instantaneous events, as shown in Fig 2c, includes mainly retweets that are forwarded from a few of solo tweets, while the time series data on non-instantaneous events, as shown in Fig 2d, contains many solo tweets and their retweets. The method presented in this paper takes into account such an effect (i.e., the diverse composition of the time series data) and the influence of multiple peaks in the time series data, and is capable of further categorizing events through distinguishing the impacts of solo tweets from those of retweets. For example, although events in Fig 2c have the similar changes in the tweet count over time (i.e., a single peak in the time series), the level of human concerns can be used as an indicator to categorize instantaneous events into small clusters, as shown in Fig 2a. That is because human concerns about events reflect the attraction of the events as well as subsequent discussions about these events.

### Heterogeneous characteristics of collective online behaviors

**Fig 3:**
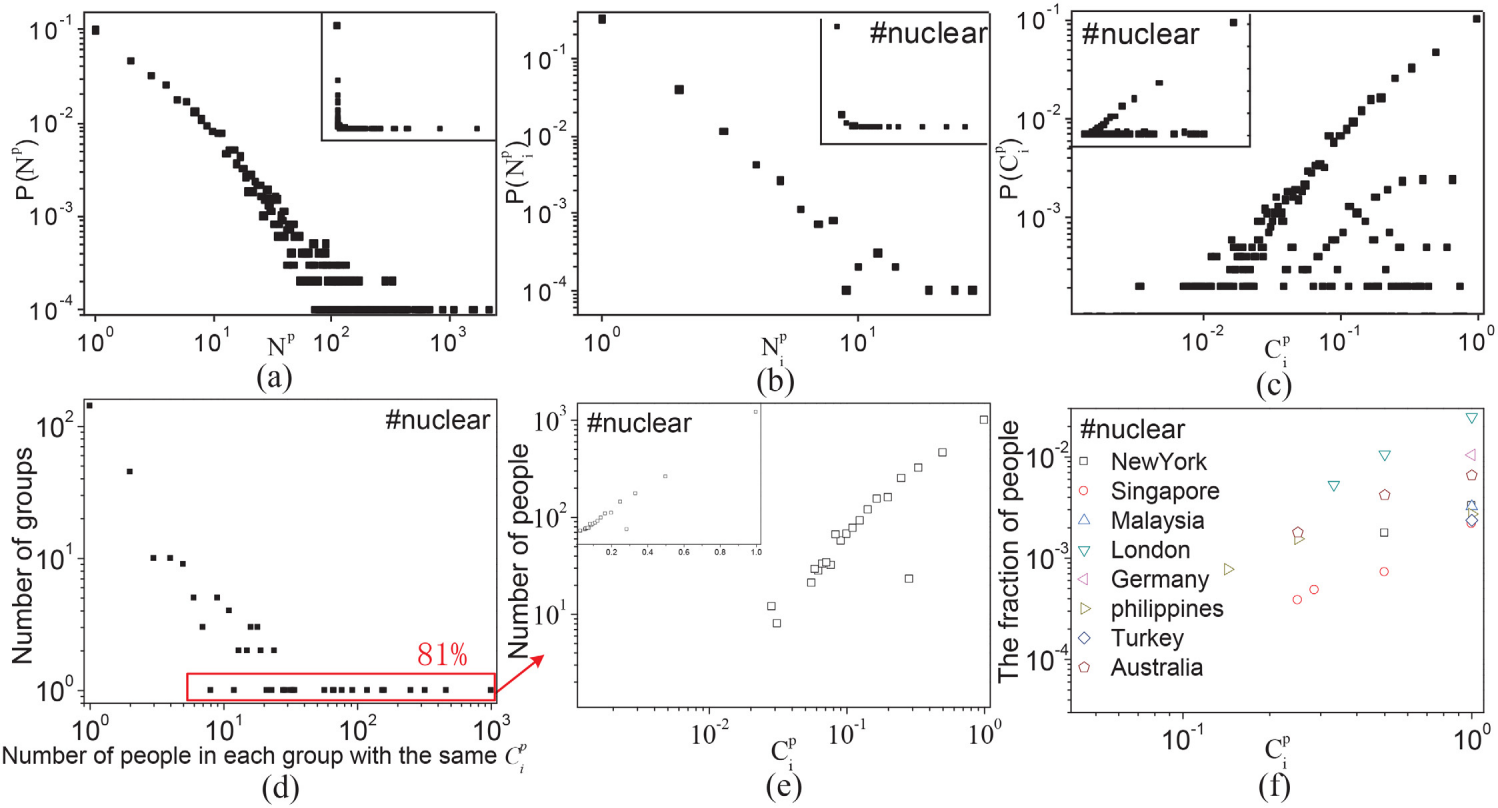
Statistical results of collective behaviors. (a) The regularity of active users and their tweets, i.e., the distribution of *N* ^*p*^ of people. (b)(c) The regularity of active users about ‘#nuclear’, i.e., the distribution of $N_{\#nuclear}^{p}$ and $C_{\#nuclear}^{p}$ of people, respectively. (d) The distribution of heterogeneous groups and the number of people in each group based on the same concern about ‘#nuclear’. A point (*x*, *y*) in (d) indicates that there is *y* different groups and each group have *x* people with the same *C* ^*p*^. (e) The distribution of *C* ^*p*^ where *y* = 1 in (d), which covers almost 81% of the total people in (c). More specifically, each dot in (e) is aggregated over all people in different regions as shown in (f). (f) The self-similarity distribution of *C* ^*p*^ (where *y* = 1) on the nuclear crisis in different regions. There exist an internal consistency of the distribution of concerns about an event among different regions. Through extending hashtag ‘#nuclear’ to term ‘nuclear’, such a nonlinear characteristic is becoming more evident as shown in Fig G in S1 Appendix.

Some population-based models, based on the mean-field theory that adopts a homogenous assumption, have been used for characterizing collective behaviors in social media [18, 35–37]. However, our previous statistical results reveal the heterogeneous human concerns about events and their durations from an individual-based perspective. In what follows, Fig 3 takes ‘#nuclear’ as an example to further illustrate such heterogeneous characteristics.

For different people, both the distributions of *N* ^*p*^ and the corresponding $N_{\#nuclear}^{p}$ follow power-law distributions as shown in Fig 3a and 3b, respectively. The distribution of $C_{\#nuclear}^{p}$ among people is shown in Fig 3c, which shows a nonlinear characteristic. Through extending hashtag ‘#nuclear’ to term ‘nuclear’, such a nonlinear characteristic is becoming more evident as shown in Fig G in S1 Appendix. Moreover, for different regions, such a nonequilibrium distribution can also be observed in Fig F(c) and Fig F(e) in S1 Appendix. More regional heterogeneous responses to an events will be discussed in the next section.

In order to observe more regularities, Fig 3d illustrates the statistical characteristics of people on ‘#nuclear’ in which each dot denotes the relationship between the number of groups with the same $C_{\#nuclear}^{p}$ and the number of individuals in each group. For example, a dot(x, y) in Fig 3d indicates that there are *y* groups and each group has *x* people. Two individuals in the same group have the same $C_{\#nuclear}^{p}$, and vice versa. Through computing the proportion of long tail (i.e., *y* = 1) to the total, we find that most of the people (near more than 80% as shown in Fig 3d and Fig F(c) in S1 Appendix) have different values of $C_{i}^{p}$ and can be divided into groups based on their $C_{i}^{p}$ as shown in Fig 3e. More specifically, this nonlinear relationship, as shown in Fig 3e, is independent of the types of events and regions of people through comparing the distribution of human concerns, in different regions, about events as shown in Fig 3f and Fig H in S1 Appendix. That is to say, for different events, people, both in the whole and in the subregions, can be divided into several groups (i.e., subpopulations) and each group has the same value of concern (i.e., $C_{i}^{p}$). And the relationship between the number of groups and the number of people in each group follows a long-tail distribution. This finding well justifies the rationality of modeling collective online responses to events through categorizing people into subpopulations and initializing the distribution of human concerns of each subpopulation in a meta-population model.

### Measuring regional responses to events

**Fig 4:**
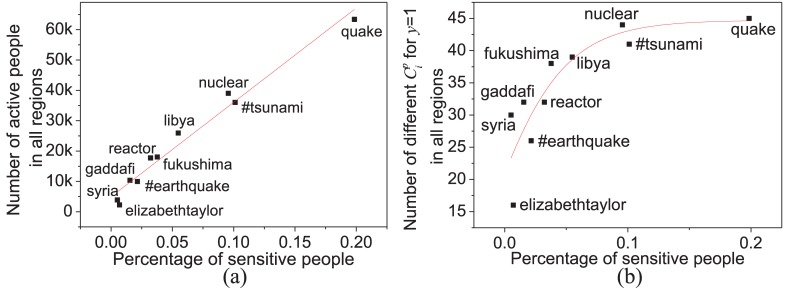
Heterogenous regional responses measured by the sensitive people. (a) The positive relationship between the percentage of sensitive people and the total amount of active people who has posted a tweet about a certain event at least once. (b) The nonlinear relationship between the percentage of sensitive people and the number of different $C_{i}^{p}$ in all regions. The more people take part in the discussions about an event, the more heterogeneity of people will appear that cause the diversity of human responses as shown in Fig H in S1 Appendix.

Fig 4a shows that there exists a positive relationship between the percentage of sensitive people and the total number of active people who post event-related tweets at least once. Here, the sensitive people refer to the people whose tweets are all related to the event (i.e., $C_{i}^{p}=1$). Therefore, the fraction of sensitive people in the dataset can be set as a benchmark to efficiently measure and distinguish the degrees of activeness among regions. If a region has a higher fraction of sensitive people than the average level, it will be more active than others. For example, Germany was more sensitive to nuclear crisis than Turkey as shown in Fig H(e) and Fig H(f) in S1 Appendix. That may be due to the fact that 24% electric in Germany was generated by nuclear power and lengthy discussions about replacing nuclear plant by renewable energy had been lasting for a long time before the nuclear reactor accident in Japan. And Germany turned off seven nuclear plants three days after the nuclear crisis occurred (see http://www.bbc.co.uk/news/business-12745899). In the mean time, Turkey was more active to social unrests in the Middle East (e.g., Libya crisis) due to the geographical relationship as shown in Fig H(g), Fig H(h) and Fig H(i) in S1 Appendix.

On the other hand, the heterogeneity of human responses to an event in the same region can be measured by the diversity of $C_{i}^{p}$ (i.e., the number of different $C_{i}^{p}$) in such a region, which is relative to the percentage of sensitive people (i.e., $C_{i}^{p}=1$) as shown in Fig 4b. Fig I in S1 Appendix further provides more illustrations to reveal heterogeneous responses to different events in the same region through comparing the percentages of sensitive people.

## Discussion

### Regional information propagation

**Fig 5:**
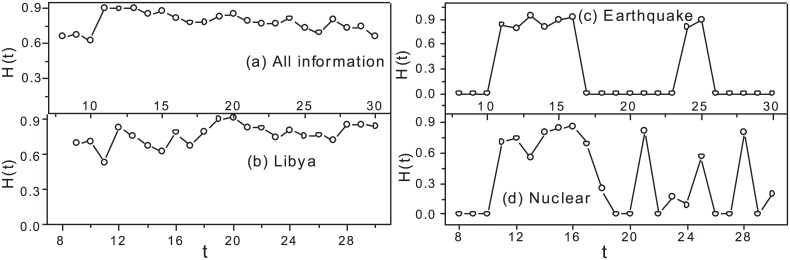
The dynamic change of regional information propagation. Generally speaking, the regional heterogeneity of information propagation is stable as shown in (a). However, there is a large fluctuation among regional information propagation in presence of different events as shown in (c)(d).

Fig 5 reveals the regional heterogeneity of information propagation based on the information entropy *H* (Eq B in S1 Appendix). First, we find that such a heterogeneity is stable most of the time as shown in Fig 5a and 5b, which is determined by one characteristic of social media (e.g., the persistence of public opinion) as discussed in [38]. For example, the Libya crisis had been lasting a whole month in social media. And the fast information dissemination and sharing mechanism of the social media help people receive more information quickly and attract people take part in such discussions. Next, for some events as shown in Fig 5c ans 5d, the entropy *H* will reduce with time due to different regional declining rates of interests in such events, which indicates that there exist heterogenous regional responses to an event. That is because people in some regions may get bored or lose interests in certain event-related information [38], and only those people who are closely connected with the event will continue paying attention to such information, which reflect the other characteristic of social media (i.e, the event-driven characteristic of information propagation with particular durations). Moreover, we note that information bulletins from public media (e.g., BBC or CNN) play an important role in regional information propagation through comparing the number of retweets that forward a tweet released by the public media, and the change of *H* value, which show another characteristic of social media, i.e., the dynamic changes of public opinion with events as shown in [38]. This finding is also supported by the latest research in [39]. For example, a tweet about ‘#*nuclear*’ was released by Reuter on March 21 that aroused a retweeted peak in the time series of tweets.

**Fig 6:**
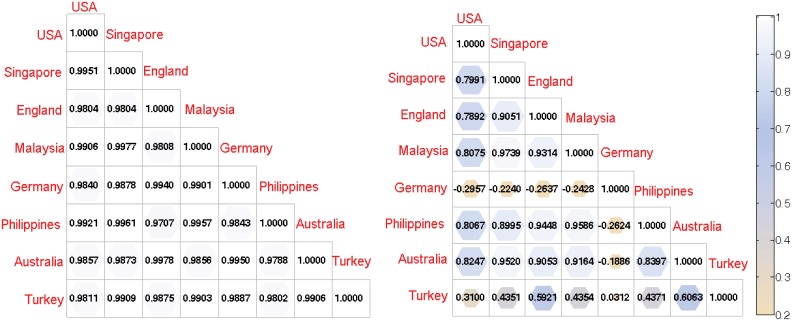
The correlation among information spreading in different regions. There exist a heterogeneous regional responses to events based on the types of events and regional profiles.

Fig 6 and Fig J in S1 Appendix show that the degrees of correlation of information propagation speed among regions, measured by the pearson coefficient, are different due to the effect of regional profiles on the regularities of responses to events. For example, there is a very strong correlation (0.9 < *r* < 1) during suddenly erupted events (e.g., Tsunami as shown in Fig 6a) due to the alarming ability of social media. On the other hand, the obvious heterogeneities are found during the non-instantaneous event (e.g., nuclear accident as shown in Fig 6b) due to the effect of regional profiles on human collective online behaviors.

### The regional evolving characteristics of human collectives

**Fig 7:**
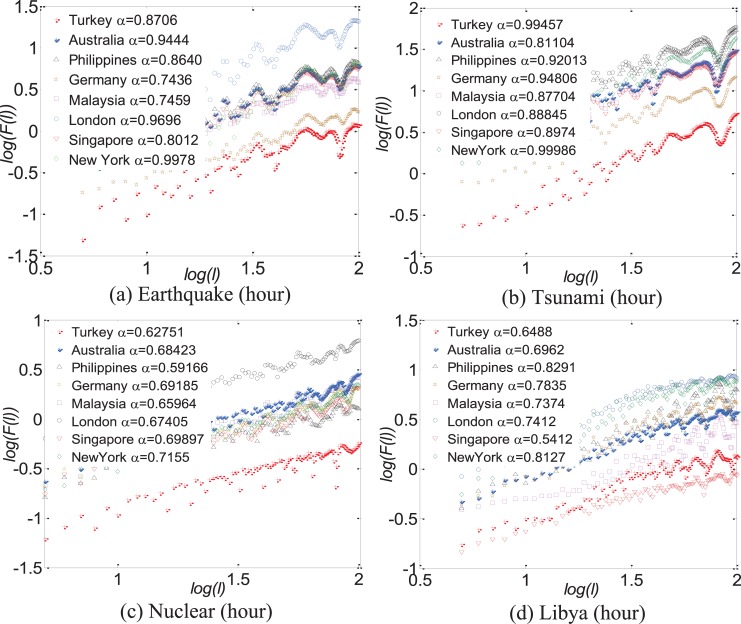
The results of collective time series measured by DFA in a log-log plot. The symbols represent the different regional responses to the same event. Similar scaling behaviors indicate that human collective online behaviors as a show follow a long-correlated self-similar process. Especially, such a long-range correlation is changed with time granularity. For example, the time series present long-range correlation by the hour regardless of regional profiles and the types of events. Yet the time series measured by the day, as shown in Fig K in S1 Appendix, may not be correlated based on the type of events.

Based on the previous empirical analysis about collected time series data on tweets, one of the most interesting phenomena is the fat-tailed characteristics following a power-law distribution. This phenomenon implies that the burst of collective online behaviors is separated by long periods of inactivity. Moreover, the scaling exponents are different according to the types of events and regional profiles. Fig 7 quantifies the long-range power-law correlations embedded in the regional self-similarity regularity based on the detrended fluctuation analysis (DFA, defined in Sec E in S1 Appendix), which is a benchmark method to quantify long-term correlations [40–42]. Results show that the scaling exponents fluctuate with regions based on the types of events, and discover several hidden patterns as follows.

First, we find that the relationship of collective time series changes with the time scales in the same region. By the hour, there exist clear long-rang correlations as shown in Fig 7, which are independent of events or regional profiles. It means that we can predict coming collective online behaviors based on the current data on human behaviors. And the larger an exponent is, the more likely we can predict collective online behaviors during an extreme event. But by the day, human collective online behaviors may not be correlated as shown in Fig K(a) and Fig K(b) in S1 Appendix. According to our understanding, there are two reasons: one is determined by the inherent characteristics of the social network (fast information dissemination and responses to events) [43]. The other is determined by the types of events. People quickly lose interests in instantaneous events (e.g., tsunami or earthquake) and will be attracted to other topics on social media.

Second, human responses to the same event show the regional self-similarity regularity through comparing the scaling exponents in different regions. For example, the scaling exponents in most of the regions are greater than 0.8 for instantaneous events (as shown in Fig 7a), and lower than 0.8 for non-instantaneous events (as shown in Fig 7b), in which the scaling exponents fluctuate within a small time interval. Although collective time series doesn’t follow a power-law distribution (i.e., the exponent is greater than 1), there also exists a self-similarity pattern among regions as shown in Fig K in S1 Appendix. The values of *α* reveal similar scaling behaviors among all regions, implying that the collective human behaviors at the systems level involves a process embodying the self-similarity and long-range correlation.

Fig L in S1 Appendix analyzes the correlations of regional concerns based on the pearson coefficient *r* (Eq D in S1 Appendix). There is a very strong correlation among regional evolution of human collective concerns, which means that the evolutional trends of regional concerns follow the same pattern during an event. This finding is consistent with Fig 3f and Fig H in S1 Appendix. That is to say, a synchronous characteristic of regional responses during events can be observed.

## Conclusion

Information relevant to decision making is critical to enhance emergency situation awareness for disaster relief. And measuring and understanding spatiotemporal characteristics of human online behaviors during extreme events can improve the emergency response to different events. This paper first reveals the distinct characteristics of human collective behaviors during events through measuring their concerns and durations, which enable us to gain a better understanding of dynamic changes in human online behaviors corresponding to different types of events. Next, we measure the heterogeneity of human responses to events from the individual and regional perspectives, which empirically confirm the heterogeneity assumption as adopted in the meta-population models for characterizing collective online responses to events. Finally, we discuss some characteristics of collective online behaviors within a region and across regions through measuring different regional speeds in information propagation based on information entropy, and modeling the self-similar evolution process of collective responses within a region based on DFA. All these findings have provided a detailed account for the nature of heterogenous human behaviors on social media in presence of extreme events.

## Supporting Information

S1 Appendix Supporting Information File. (PDF)

## References

[1] Yin J, Lampert A, Cameron M, Robinson B, Power R (2012) Using social media to enhance emergency situation awareness. IEEE Intell Syst 27: 52–59. 10.1109/MIS.2012.6

[2] Middleton SE, Middleton L, Modafferi S (2014) Real-time crisis mapping of natural disasters using social media. IEEE Intell Syst 29: 9–17. 10.1109/MIS.2013.126

[3] Mendonca D, Wallace WA (2007) A cognitive model of improvisation in emergency management. IEEE Trans Syst Man Cybern A 374: 547–561. 10.1109/TSMCA.2007.897581

[4] Hua T, Lu CT, Ramakrishnan N, Chen F, Arredondo J, Mares D, Summers K (2013) Analyzing civil unrest through social media. Computer 46: 80–84. 10.1109/MC.2013.442

[5] Boyle MP, Schmierbach M, Armstrong CL, McLeod DM, Shah DV, Pan ZD (2004) Information seeking and emotional reactions to the September 11 terrorist attacks. Journal Mass Commun Q 81: 155–167. 10.1177/107769900408100111

[6] Bakshy E, Rosenn I, Marlow C, Adamic L (2012) The role of social networks in information diffusion. In: Mille A, Gandon FL, Misselis J, Rabinovich M, Staab S, editors. WWW2012: Proceedings of the 21st International World Wide Web Conference; 2012 Apr 16–20; Lyon, France. New York: ACM; 2012. p. 519–528. 10.1145/2187836.2187907

[7] Jones J, Salathe M (2009) Early assessment of anxiety and behavioral response to novel swine-origin influenza a H1N1. PLoS ONE 4: e8032 10.1371/journal.pone.0008032 19997505 PMC2779851

[8] Golder SA, Macy MW (2011) Diurnal and seasonal mood vary with work, sleep, and day length across diverse cultures. Science 333: 1878–1881. 10.1126/science.1202775 21960633

[9] Lansdall-Welfare T, Lampos V, Cristianini N (2002) Effects of the recession on public mood in the UK. In: Mille A, Gandon FL, Misselis J, Rabinovich M, Staab S, editors. WWW2012: Proceedings of the 21st International World Wide Web Conference (Companion Volume); 2012 Apr 16–20; Lyon, France. New York: ACM; 2012. p. 1221–1226. 10.1145/2187980.2188264

[10] Zhao ZD, Yang ZM, Zhang ZK, Zhou T, Huang ZG, Lai YC (2013) Emergency of scaling in human-interest dynamics. Sci Rep 3: 3472 10.1038/srep03472 24326949 PMC3858797

[11] Zhao ZD, Cai SM, Lu Y (2015) Non-Markovian character in human mobility: Online and offline. Chaos 25: 063106 10.1063/1.4922302 26117100

[12] Grabowicz PA, Ramasco JJ, Moro E, Pujol J, Eguiluz VM (2012) Social features of online networks: the strength of intermediary ties in online social media. PLoS ONE 7: e29358 10.1371/journal.pone.0029358 22247773 PMC3256152

[13] Bollen J, Goncalves B, Ruan G, Mao H (2011) Happiness is assortative in online sosical networks. Artif Life 17: 237–251. 10.1162/artl_a_00034 21554117

[14] Sakaki T, Okazaki M, Matsuo Y (2013) Tweet analysis for real-time event detection and earthquake reporting system development. IEEE Trans Knowl Data Eng 25: 919–931. 10.1109/TKDE.2012.29

[15] Signorini A, Segre AM, Polgreen PM (2001) The use of Twitter to track levels of disease activity and public concern in the U.S. during the influenza A H1N1 pandemic. PLoS ONE 6: e19467 10.1371/journal.pone.0019467 PMC3087759 21573238

[16] Shneiderman B, Preece J, Pirolli P (2011) Realizing the value of social media requires innovative computing research. Commun ACM 54: 34–37. 10.1145/1995376.1995389

[17] Preis T, Moat HS, Stanley HE (2013) Quantifying trading behavior in financial markets using Google Trends. Sci Rep 3: 1684 10.1038/srep01684 23619126 PMC3635219

[18] Matsubara Y, Sakurai Y, Prakash BA, Li L, Faloutsos C (2012) Rise and fall patterns of information diffusion: model and implications. In: Yang Q, Agarwal D, Pei J, editors. KDD2012: Proceedings of the 18th ACM SIGKDD International Conference on Knowledge Discovery and Data Mining; 2012 Aug 12–16; Beijing, China. New York: ACM; 2012. p. 6–14. 10.1145/2339530.2339537

[19] Sasahara K, Hirata Y, Toyoda M, Kitsuregawa M, Aihara K (2013) Quantifying collective attention from tweet stream. PLoS ONE 8: e61823 10.1371/journal.pone.0061823 23637913 PMC3640043

[20] Sano Y, Yamada K, Watanabe H, Takayasu H, Takayasu M (2013) Empirical analysis of collective human behavior for extraordinary events in the blogosphere. Phys Rev E 87: 012805 10.1103/PhysRevE.87.012805 23410386

[21] Crane R, Sornette D (2008) Robust dynamic classes revealed by measuring the response function of a social system. Proc Natl Acad Sci U S A 105: 15649–15653. 10.1073/pnas.0803685105 18824681 PMC2572957

[22] Abhik D, Toshniwal D (2013) Sub-event detection during natural hazards using features of social media data. In: Carr L, Laender AHF, Loscio BF, King I, Fontoura M, Vrandecic D, Aroyo L, Oliveira JPM, Lima F, Wilde E, editors. WWW2013: Proceedings of the 22nd International World Wide Web Conference (Companion Volume); 2013 May 13–17; Rio de Janeiro, Brazil. New York: ACM; 2013. p. 783–788.

[23] Yang J, Leskovec J (2011) Patterns of temporal variation in online media. In: King I, Nejdl W, Li H, editors. WSDM2011: Proceedings of the 4th ACM International Conference on Web Search and Data Mining; 2011 Feb 9–12; Hong Kong, China. New York: ACM; 2011. p. 177–186. 10.1145/1935826.1935863

[24] Lehmann J, Goncalves B, Ramasco J, Cattuto C (2012) Dynamical classes of collective attention in Twitter. In: Mille A, Gandon FL, Misselis J, Rabinovich M, Staab S, editors. WWW2012: Proceedings of the 21st International World Wide Web Conference; 2012 Apr 16–20; Lyon, France. New York: ACM; 2012. p. 251–258. 10.1145/2187836.2187871

[25] Xu ZH, Zhang Y, Wu Y, Yang Q (2012) Modeling user posting behavior on social media. In: Hersh WR, Callan J, Maarek Y, Sanderson M, editors. SIGIR2012: Proceedings of 35th International ACM SIGIR Conference on Research and Development in Information Retrieval; Aug 12–16; Portland, OR, USA. New York: ACM; 2012. P. 545–554. 10.1145/2348283.2348358

[26] Weng L, Flammini A, Vespignani A, Menczer F (2012) Competition among memes in a world with limited attention. Sci Rep 2: 335 10.1038/srep00335 22461971 PMC3315179

[27] Twitter REST API. Available at https://dev.twitter.com/rest/public.

[28] Wei J, Zhao D, Liang L (2009) Estimating the growth models of news stories on disasters. J ASSOC INF SCI TECH 60: 1741–1755. 10.1002/asi.21109

[29] Tufekci Z. Big Data: pitfalls, methods and concepts for an emergent filed. Available from: http://ssrn.com/abstract=2229952.

[30] Gao C, Liu JM (2012) Clustering-based media analysis for understanding human emotional reactions in an extreme event. In: Chen L, Felfernig A, Liu JM, Ras ZW, editors. ISMIS2012: Proceedings of 20th International Symposiumon Methodologies for Intelligent Systems; 2012 Dec 4–7; Macau, China. Berlin: Springer; 2012. p. 125–135. 10.1007/978-3-642-34624-8_15

[31] Newman MEJ (2004) Fast algorithm for detecting community structure in networks. Phys Rev E 6: 066133 10.1103/PhysRevE.69.066133 15244693

[32] Onnela JP, Reed-Tsochas F (2010) Spontaneous emergence of social influence in online systems. Proc Natl Acad Sci U S A 43: 18375–18380. 10.1073/pnas.0914572107 PMC2972979 20937864

[33] Kleinberg J (2008) The convergence of social and technological networks. Commun ACM 51: 66–72. 10.1145/1400214.1400232

[34] Clauset A, Shalizi CR, Newman MEJ (2009) Power-law distribution in empirical data. SIAM Rev 51: 661–703. 10.1137/070710111

[35] Radinsky K, Svore K, Dumais S, Teevan J, Bocharov A, Horvitz E (2012) Modeling and predicting behavioral dynamics on the web. In: Mille A, Gandon FL, Misselis J, Rabinovich M, Staab S, editors. WWW2012: Proceedings of the 21st International World Wide Web Conference; 2012 Apr 16–20; Lyon, France. New York: ACM; 2012. p. 599–608. 10.1145/2187836.2187918

[36] Myers S, Zhu C, Leskovec J (2012) Information diffusion and external influence in networks. In: Yang Q, Agarwal D, Pei J, editors. KDD2012: Proceedings of the 18th ACM SIGKDD International Conference on Knowledge Discovery and Data Mining; 2012 Aug 12–16; Beijing, China. New York: ACM; 2012. p. 33–41. 10.1145/2339530.2339540

[37] Ribeiro B (2014) Modeling and predicting the growth and death of membership-based websites. In: Chung CW, Broder AZ, Shim K, Suel T, editors. WWW2014: Proceedings of the 23rd International World Wide Web Conference; 2014 Apr 7–11; Seoul, Republic of Korea. New York: ACM; 2014. p. 653–664. 10.1145/2566486.2567984

[38] Gong K, Tang M, Shang MS, Zhou T (2012) Empirical study on Spatio-temporal evolution of online public opinion. Acta Phys Sin 61(9): 098901.

[39] Kim M, Newth D, Christen P (2014) Trends of news diffusion in social media based on crowd phenomena. In: Chung CW, Broder AZ, Shim K, Suel T, editors. WWW2014: Proceedings of the 23rd International World Wide Web Conference (Companion Volume); 2014 Apr 7–11; Seoul, Republic of Korea. New York: ACM; 2014. p. 753–758.

[40] Rybski D, Buldyrev SV, Havlin S, Liljeros F, Makse HA (2012) Communication activity in a social network: relation between long-term correlations and inter-event clustering. Sci Rep 2: 560 10.1038/srep00560 22876339 PMC3413962

[41] Cai SM, Fu ZQ, Zhou T, Gu J, Zhou PL (2009) Scaling and memory in recurrence intervals of internet traffic. EUROPHYS LETT 87: 68001 10.1209/0295-5075/87/68001

[42] Zhao ZD, Cai SM, Huang JM, Fu Y, Zhou T (2012) Scaling behavior of online human activity. Europhys Lett 100: 48004 10.1209/0295-5075/100/48004

[43] Mills A, Chen R, Lee J, Rao HR (2009) Web 2.0 emergency applications: how useful can twitter be for emergency response? Journal of Information Privacy and Security 5: 3–26. 10.1080/15536548.2009.10855867

