# Correction: Uncovering Spatiotemporal Characteristics of Human Online Behaviors during Extreme Events

**DOI:** 10.1371/journal.pone.0144666

**Published:** 2015-12-04

**Authors:** The PLOS ONE Staff

The following information is missing from the Funding section: This work was supported by the National Natural Science Foundation of China (Nos. 61402379, 61403315), Natural Science Foundation of Chongqing (Nos. cstc2012jjA40013, cstc2013jcyjA40022) and the open project program of Key Laboratory of Symbolic Computation and Knowledge Engineering of Ministry of Education, Jilin Universty. The funders played no role in the study design, data collection and analysis, decision to publish, or preparation of the manuscript.

The publisher apologizes for the error.
