# Supplementary material for: Uncovering Spatiotemporal Characteristics of Human Online Behaviors during Extreme Events
Source: PLoS One. 2015 Oct 22;10(10):e0138673. doi: 10.1371/journal.pone.0138673 (PMC4619611; doi:10.1371/journal.pone.0138673)
Supplement: S1 Appendix — (PDF) [file pone.0138673.s001.pdf]

Uncovering Spatiotemporal Characteristics of Human Online Behaviors during Extreme Events - Supplementary Information

Chao Gao[1], Jiming Liu[2,*]

1 College of Computer and Information Science, Southwest University, Chongqing, China
2 Department of Computer Science, Hong Kong Baptist University, Kowloon Tong, Hong Kong

*** jiming@comp.hkbu.edu.hk**

Abstract

In response to an extreme event, individuals on social media demonstrate interesting behaviors in information seeking and sharing, depending on their backgrounds. Existing studies have attempted to analyze and understand the regularities of human responses during an extreme event, e.g., their spatiotemporal patterns. However, most of them focus on the general patterns of human collective online activities from a population perspective. In reality, individuals with different backgrounds, e.g., living in different geographic locations, will behave differently. Therefore, it remains to be a crucial issue for us to quantitatively understand human collective online behaviors during extreme events, especially from an individual perspective. Due to the difficulty in measuring individual behaviors and the lack of datasets from which the underlying dynamical processes may be inferred, it is more challenging for us to probe into the dynamics of human collective online behaviors. By making use of the large-scale datasets of posts and search queries collected from Twitter and GoogleTrends during various events, it is now possible for us to analyze individuals' online behaviors and then reveal distinct characteristics (i.e., heterogeneous spatiotemporal patterns) of collective online responses to extreme events.

A. Dataset and Methods

Two different data sources, Twitter and GoogleTrends, are used to analyze human online behaviors in which Twitter represents collective information sharing during events and GoogleTrends represents collective information seeking behaviors, respectively. Table A shows the general statistics about the Twitter data and Fig A plots the time series data that reflects the temporal characteristics of collective behaviors during events. Specifically, a keyword-based analysis is performed in this paper for identifying an event. Based on hashtags in the same tweets, a hashtag network is constructed, which is a weighted network $G =< N, E >$. Each edge $(i, j) \in E$ is associated with a weight w_{ij}. Since our dataset includes 4,972 distinct hashtags and parts of hashtags only appear a few times, we select the most popular hashtags by restricting $w_{ij} > 100$. After that, a clustering-based analysis, introduced in [1], is first used to categorize hashtags into different groups based on the modularity

Q [2]. More specifically, the modularity Q is defined as $Q = \sum_k (c_{kl} - a_k^2)$. At the beginning, each hashtag is regarded as a single community. c_{kl} is defined as the fraction of edges that connect hashtag in group C_k to others in group C_l, and $a_k = \sum_l c_{kl}$. This greedy algorithm aims to maximize Q at each step through merging different small clusters into a big one, i.e., maximizing $\triangle Q = 2(c_{kl} - a_k a_l)$ at each step. When Q reaches to the maximum value, the best division is obtained. Hashtags with cluster label are grouped together based on the co-occurrence of hashtags appearing in a tweet. Based on such clustering analysis, some core-periphery structures are detected from the hashtag network as shown in Fig B(a), Fig B(b) and Fig B(c), more analysis can be found in [3]. We select those core hashtags to represent events and search related keywords from GoogleTrends for comparing the event classification results and estimating the effect of solo tweets.

Most of the studies on uncovering spatiotemporal characteristics of human online behaviors only consider the total amount of tweets and don't address the contribution of individual behaviors (i.e., corresponding to different concerns on events) to overall influences [4, 5]. This paper defines two indicators to measure collective behaviors from the perspective of individual behaviors, i.e., human concerns about events and durations of such concerns. More specifically, the collective online concern is measured by the exponent α_c drawn from the cumulative distribution of solo tweets about an event. And the duration of such a concern is measured by the exponent α_T drawn from the distribution of new incremental tweets about an event, as shown in Fig C. Based on the proposed two indicators, we can observe clear heterogeneous characteristics of human online behaviors, which can in turn be used to categorize events into groups as shown in Fig D.

B. The importance of solo tweets on Twitter

In this section, we illustrate the importance of solo tweets on Twitter as well as its implication to events classification. Specifically, through comparing with the classification result with that of GoogleTrends, we can confirm the speculation: solo tweets can further reflect human collective online concerns about events.

Based on the dynamic time series data from online social media, researchers have adopted some indicators to categorize human collective online behaviors during events. For example, the proportion of a peak value (measured by the highest volume) in the time series to the total amount of tweets has been used for event classification [4, 5]. However, this indicator considers only the global behaviors through computing the proportion of 'before', 'during' and 'after' the peak in the time series, and doesn't address the contribution of individual behaviors to overall influences. In what follows, a case study is given to reveal the effect of individual behaviors on event classification based on the traditional peak analysis [5].

Through searching the same event-related keywords on Twitter and GoogleTrends, Fig A plots the dynamic changes of time series data about events in which $n_i(t)$ denotes the total number of human activities (i.e., number of tweets, as shown in Fig A(a), and search volume index in GoogleTrends, as shown in Fig A(b)) about event i on day t. If the peak value of activities appears on day t_0, we take t_0 as a center point in the time series. Particularly, the proportion of peak value during one week (i.e., the size of sliding window is 7) is defined as f_{peak}, and the total number of activities 'before' and 'after' the peak is defined as f_{before} and f_{after}, respectively, as follows:

$$\begin{cases} f_{before} = \sum\limits_{t_0-3 \le t < t_0} n(t)/N \\ f_{peak} = n(t_0)/N \qquad\qquad where \quad N = \sum_{t_0-3}^{t_0+3} n(t) \\ f_{after} = \sum\limits_{t_0 < t \le t_0+3} n(t)/N \end{cases} \tag{A}$$

Based on the proportion of the peak value to the amount of $n_i(t)$, Fig E illustrates that events can be grouped into clusters. However, the classification results of two datasets are not consistent when comparing Fig E(a) with Fig E(b), which is caused by the different contribution of individual behaviors to overall collective activities. Based on the handbooks of GoogleTrends (https://support.google.com/trends/answer/4355213?hl=en) and Twitter (https://dev.twitter.com/overview/documentation), Fig A(b) eliminates repetitive queries from the same user over a short period of time, which emphasizes the contribution of each people. On the other hand, Fig A(a) is plotted based on the total amount of tweets about events, which uniformizes the contribution of individual behaviors. The statistics show that the final time series of Fig A(a) is composed of two parts: retweets and solo tweets. If we take human behaviors on Twitter as the activities in a complex system [6, 7], retweets can be seen as the endogenous activities of such a system, i.e., a tweet, which contains a certain type of information, is retweeted by others repeatedly over a short period of time (as shown in Fig F(d)); while, solo tweets are exogenous activities for such a system, i.e., some original tweets, which can be seen as new information sources, are injected into Twitter. Although some epidemic-based models have been used to explore the coupling effects of endogenous and exogenous impacts on collective behaviors on Twitter [8, 9], the latest empirical study has illustrated the difference between the conclusion, as drawn from the epidemic-based information propagation model, and the statistical result of collective attention from the social media [10]. More specifically, the empirical study has proven that solo tweets are unique to collective attention, rather than retweets [10].

According to the above viewpoint, Table B takes some examples to separate solo tweets from the final time series data in Fig A(a) and compute the proportion of solo tweets to all tweets (defined as $P - solo$). By comparing the $P - solo$ of hashtags and the corresponding classification results in Fig E(b), we find that hashtags with the similar values of $P - solo$ are clustered into the same group based on GoogleTrends. This means that solo tweets play an important role in identifying the type of events. Based on this finding, we propose a new method to categorize extreme events based on the distribution of solo tweets that can further reflect the diversity of human opinions and concerns about events.

C. Heterogeneous spatial characteristics of human collective online behaviors

Figure F illustrates heterogeneous spatial characteristics of collective online behaviors. First of all, people in the same region have different responses to events, as shown in Fig F(a), Fig F(b) and Fig G(c). On the other hand, people in different regions also have distinct responses to the same event. Taking 'fukushima' as an example, Fig F(c) and Fig F(e) plot the distributions of $N^p_{fukushima}$ and the corresponding N^p for different cities, which show that most of the tweets and retweets were released by only a few of cities that were most concerned about this event. Statistical results reveal that responses of cities to an event are nonequilibrium. Taking 'nuclear' as an example, people are clustered into groups based on their concerns about events, as

shown in Fig G(b). This conclusion is independent of events and regions, as shown in Fig G(c) and Fig H, which can justify the heterogeneous assumption adopted in the meta-population models (i.e., population is divided into various homogeneous groups, where each group has its own reaction-diffusion process) for characterizing and analyzing human collective online behaviors.

Figure H provides more examples to illustrate heterogeneous regional responses to events. From these statistical results, we find that there exists an internal consistency of the distribution of concerns about events among regions. Specifically, the dots of 'all' in Fig H(d) and Fig H(g) indicate the fraction of people who post certain tweets relative to all users in the dataset. The fraction of sensitive people in a region (i.e., C^p =1, denoted as the solid node) is regarded as the benchmark value to efficiently measure the degrees of activeness among regions. Based on this value, Fig I compares regional activeness in response to the events. Through comparing the percentages of sensitive people in different regions with this benchmark value, we can observe the activeness of a region during an event. Through these statistical results, we conclude that the human collective online behaviors during extreme events are mainly affected by the perceived risk about an event. This risk is related to the geographical relationship and historical experiences. For example, people in the South Asia were shown to be more sensitive to the earth quake and tsunami due to their tragic memory about Indian Ocean tsunami 2004, as well as to nuclear crisis due to the geographical influences of potential radiation leaks. Germany was more sensitive to the nuclear crisis than others because the discussion about whether or not turning off its nuclear plants had been lasting for a long time before the nuclear reactor accident in Japan. The social unrests in Libya and Syria attracted more attention in Turkey than others due to the geographical and historical relationship. On the other hand, due to the bias of social media analysis on data sampling [11] , the point of 'London' is always higher than others.

D. Heterogeneous regional information propagation

In order to reveal the heterogeneous information propagation among regions, we use information entropy (H, defined in Eq B [12]) to measure the propagation rate of event-related information.

$$H(t) = -\frac{1}{\log M} \sum_{r} p_r(t) \log p_r(t) \tag{B}$$

where M is the total number of regions and $r \in [1, M]$. $p_r(t) = \Delta n_r(t)/\Delta n(t)$ denotes the proportion of new increment tweets from region r relative to all new increment tweets at time t, $\sum_r^M p_r(t) = 1$. The heterogeneity of information propagation among regions is the highest (i.e., $H = 0$) if event-related tweets only appear in one region. With the increment of regions taking part in event-related discussions, the heterogeneity will decline (i.e., H will increase). When a certain event-related information propagates in all regions (i.e., satisfaction with the condition of the maximum entropy), $H = 1$.

Figure J compares the correlations of information propagation speed among regions based on the pearson coefficient for understanding the effect of regional profiles on the regularities of responses to events. The correlation analysis in Fig J(a) shows that the degrees of correlation are different among regional information propagation speeds due to the heterogeneous human responses to events. For example, there is a very strong correlation ($0.9 < r < 1$) during suddenly erupted events (e.g., Earthquake or Tsunami as shown in Fig J(b) and Fig J(c)) due to the alarming ability of social media. On the

other hand, the obvious heterogeneities are found during the instantaneous event (e.g., nuclear accident or Libya crisis as shown in Fig J(d) and Fig J(e)) due to the effect of regional profiles on human collective online behaviors.

E. The regional evolving characteristics of human collective online behaviors

In order to measure the regional heterogeneity, we apply a typical nonstationary time series analysis method, i.e., detrended fluctuation analysis (DFA) [13–15], to quantify the long-range power-law correlations embedded in the regional self-similarity regularity. DFA provides a feasible scaling exponent to represent the correlation characteristic of the time series.

Given a time series of collective behaviors $n_i(t)$ about an event i, where $t \in [1, T]$ and T is the length of the series, we first filter the effect of average value of the series through computing the accumulated deviation $y_i(k) = \sum_{t=1}^{k} [n_i(t) - <n_i>]$ where $<n_i>$ is the mean. And the entire time series $y_i(k)$ of length T is divided into nonoverlapping boxes of equal length L. Then, a least squares straight-line is calculated in each box of length L that represents the trend of this box. In this paper, a 2nd-order polynomial function $y_i^l(k)$ is used to fit to the data. After that, we detrend the integrated time series (i.e., $y_i(k)$) by subtracting the local trend (i.e., $y_i^l(k)$) in each box and calculate the variance of the residual time series based on Eq C.

Through computing over all time scales (i.e., box sizes l) and repeating the detrending procedure based on Eq C, we can characterize the relationship between the average fluctuation ($F(l)$) and the box size (l). They follow a power-law relationship, $F(l) \sim l^{\alpha}$. Under such conditions, the fluctuations of given time series can be characterized by a scaling exponent (i.e., α). Specifically, the time series is correlated, no correlation and anticorrelated if $\alpha > 0.5$, $\alpha = 0.5$ and $\alpha < 0.5$, respectively.

$$F(l) = \sqrt{\frac{1}{T} \sum_{k=1}^{T} [y_i(k) - y_i^l(k)]^2} \tag{C}$$

Moreover, Fig L reveals the correlation of regional evolution based on the pearson coefficient r, as shown in Eq D. There is a very strong correlation among regional evolution trends of human collective online behaviors, which means that the evolutional trends of human collective online behaviors among regions follow a similar pattern during an event. That is to say, a synchronous characteristic of regional responses during events can be observed to a certain extent.

$$r_{ab} = \frac{\sum_{t=1}^{T} (x_a(t) - <x_a>)(x_b(t) - <x_b>)}{T \cdot S_a \cdot S_b} \tag{D}$$

where $x_a(t)$ denotes the total amount of tweets in region a at time t. $<x_a>$ and S_a denote the average number and standard deviation of tweets in region a during T days, respectively.

References

1. Gao C, Liu JM (2012) Clustering-based media analysis for understanding human emotional reactions in an extreme event. In: Chen L, Felfernig A, Liu JM, Ras ZW, editors. ISMIS2012: Proceedings of 20th International Symposiumon Methodologies for Intelligent Systems; 2012 Dec 4-7; Macau, China. Berlin: Springer; 2012. p. 125–135. doi:10.1007/978-3-642-34624-8_15

2. Newman MEJ (2004) Fast algorithm for detecting community structure in networks. Phys Rev E 6: 066133. doi:10.1103/PhysRevE.69.066133

3. Shi N, Gao C, Zhang ZL, Zhong L, Huang JJ (2013) The spontaneous behavior in extreme events: A clustering-based quantitative analysis. In: Yao M, Wang W, Zaiane O, Cao LB, Wu ZH, Motoda H, editors. ADMA2013: Proceedings of 9th International Conference on Advanced Data Mining and Applications; 2013 Dec 14-16; Hangzhou, China. Berlin: Springer; 2013. p. 336–347. doi:10.1007/978-3-642-53914-5_29

4. Crane R, Sornette D (2008) Robust dynamic classes revealed by measuring the response function of a social system. Proc Natl Acad Sci U S A 105: 15649–15653. doi:10.1073/pnas.0803685105

5. Lehmann J, Goncalves B, Ramasco J, Cattuto C (2012) Dynamical classes of collective attention in Twitter. In: Mille A, Gandon FL, Misselis J, Rabinovich M, Staab S, editors. WWW2012: Proceedings of the 21st International World Wide Web Conference; 2012 Apr 16-20; Lyon, France. New York: ACM; 2012. p. 251–258. doi:10.1145/2187836.2187871

6. Onnela JP, Reed-Tsochas F (2010) Spontaneous emergence of social influence in online systems. Proc Natl Acad Sci U S A 43: 18375–18380. doi:10.1073/pnas.0914572107

7. Sano Y, Yamada K, Watanabe H, Takayasu H, Takayasu M (2013) Empirical analysis of collective human behavior for extraordinary events in the blogosphere. Phys Rev E 87: 012805. doi:10.1103/PhysRevE.87.012805

8. Myers S, Zhu C, Leskovec J (2012) Information diffusion and external influence in networks. In: Yang Q, Agarwal D, Pei J, editors. KDD2012: Proceedings of the 18th ACM SIGKDD International Conference on Knowledge Discovery and Data Mining; 2012 Aug 12-16; Beijing, China. New York: ACM; 2012. p. 33–41. doi:10.1145/2339530.2339540

9. Moran J, Cordaro F (2009) Understanding the hit-rate dynamics of a large website with an agent-based model. In: Sierra C, Castelfranchi C, Decker KS, Sichman JS, editors. AAMAS2009: Proceedings of the 8th International Conferences on Autonomous Agents and Multiagent Systems; 2009 May 10-15; Budapest Hungary. New York: ACM; 2009. p. 105–109.

10. Sasahara K, Hirata Y, Toyoda M, Kitsuregawa M, Aihara K (2013) Quantifying collective attention from tweet stream. PLoS ONE 8: e61823. doi:10.1371/journal.pone.0061823

11. Tufekci Z. Big Data: pitfalls, methods and concepts for an emergent filed. Available from: http://ssrn.com/abstract=2229952.

12. Colizza V, Barrat A, Barthelemy M, Vespignani A (2006) The role of the airline transportation network in the prediction and predictability of global epidemics. Proc Natl Acad Sci U S A 103: 2015–2020. doi:10.1073/pnas.0510525103

13. Rybski D, Buldyrev SV, Havlin S, Liljeros F, Makse HA (2009) Scaling laws of human interaction activity. Proc Natl Acad Sci U S A 106: 12640–12645. doi:10.1073 / pnas.0902667106

14. Rybski D, Buldyrev SV, Havlin S, Liljeros F, Makse HA (2012) Communication activity in a social network: relation between long-term correlations and inter-event clustering. Sci Rep 2: 560. doi:10.1038/srep00560

15. Cai SM, Fu ZQ, Zhou T, Gu J, Zhou PL (2009) Scaling and memory in recurrence intervals of internet traffic. EUROPHYS LETT 87: 68001. doi:10.1209/0295-5075/87/68001

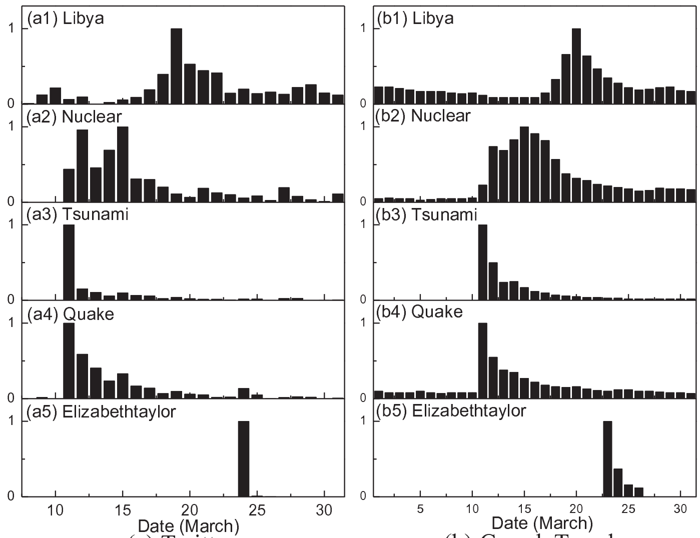

(a) Twitter (b) GoogleTrends

Figure A. The *temporal* changes of collective behaviors during different events. The Twitter data is normalized between 0 and 1. These figures show the heterogeneous characteristics of human behaviors in the temporal scale. Specifically, different dynamic processes of collective behaviors are found in two datasets because individual differences are considered in GoogleTrends.

Table A. The general statistics about the Twitter dataset

Number of Tweets	513441
Number of Tweets with hashtags	215227
Number of hashtags	4972
Number of users	197800

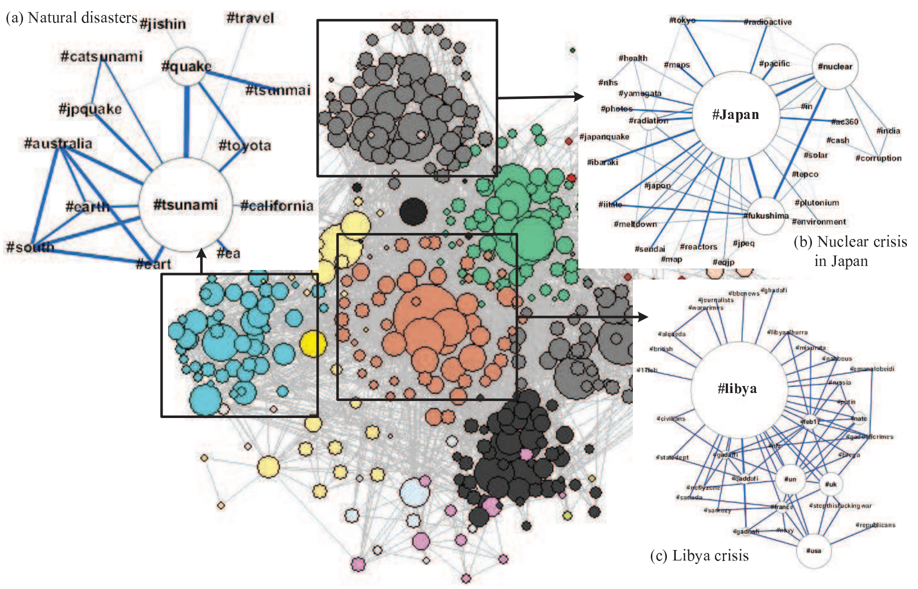

Figure B. The illustration of whole hashtags network and subgraphs of three events.

Table B. The proportion of solo tweets to all tweets on the peak day (%)

**Hashtag**	**P-solo**
Burma	2.17
Quake	2.45
Tsunami	2.80
Karangetang	2.87
Fukusihma	3.16
Nuclear	3.33
Reactors	3.40
Earthquake	4.19
Libya	7.76
Qaddafi	9.00
Libyan	9.04
Deraa	9.94

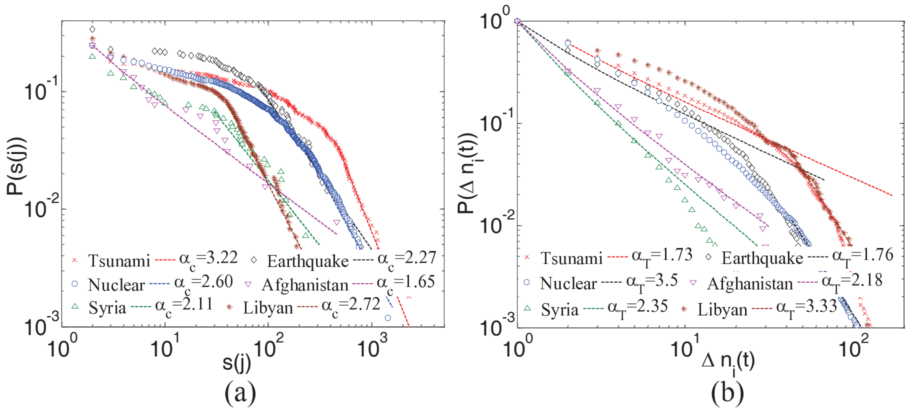

Figure C. Illustrations about the (a) distribution of retweeted network of a hashtag and its exponent α_c, and (b) distribution of incremental tweets about a hashtag and its exponent α_T. The exponent is estimated by KS statistical test. If some hashtags don't pass the estimation, we use the same term to replace such hashtags through deleting '#'. If such a word cannot pass the estimation still, these hashtags will be ignored.

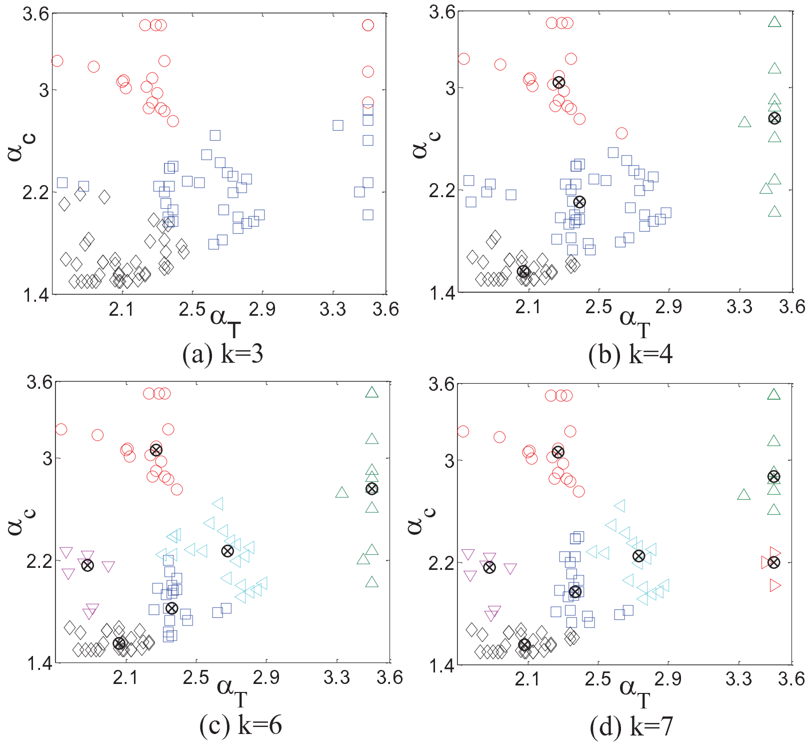

Figure D. The clustering result of events based on k-means algorithm. Based on the exponents of the distribution of collective concerns about events and the distribution of durations of such concerns, events are grouped into different clusters.

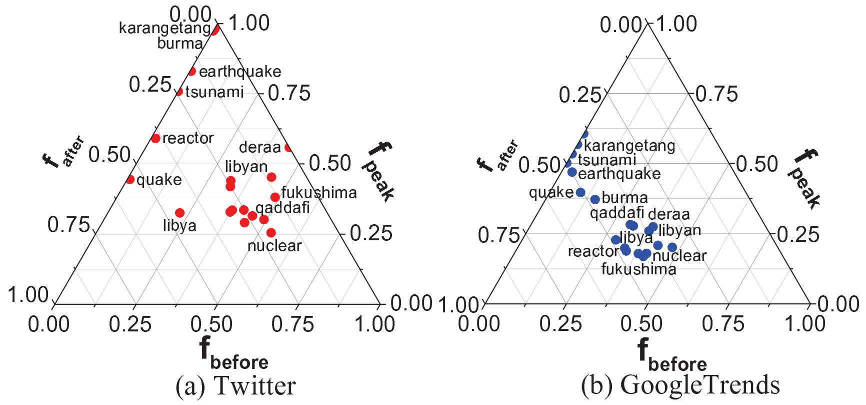

Figure E. Events are categorized into different groups based on the time series of collective activities extracted from (a) Twitter and (b) GoogleTrends, as shown in Fig A(a) and (b), respectively. The inconsistent classification between two sources are caused by the different component of two datasets. The data of GoogleTrends eliminates repeated queries from the single user over a short period of time, while the data of Twitter includes all tweets over a period of time.

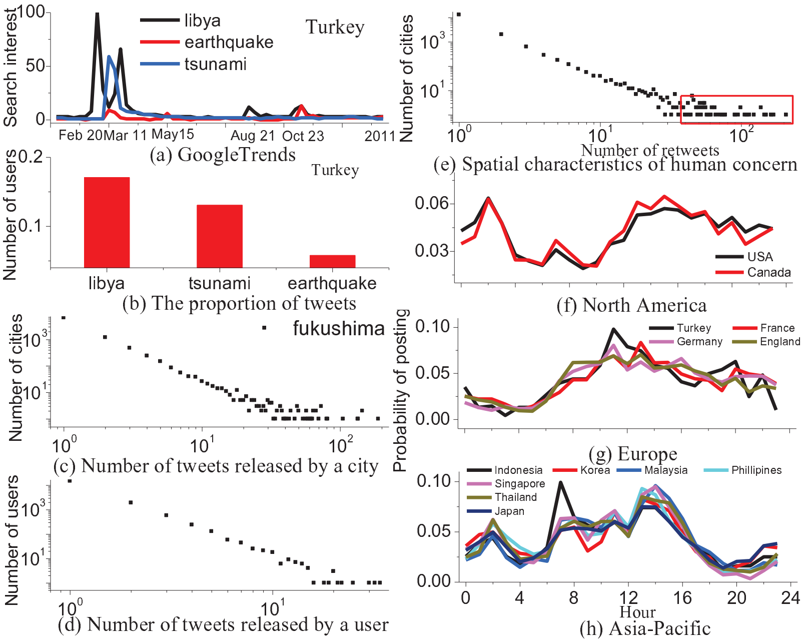

Figure F. The *spatial* heterogeneous characteristics of collective behaviors during different events based on GoogleTrends and Twitter. (a)(b) compare collective behaviors during three different events in Turkey, which demonstrate the heterogeneous concern of people in the same region. (c)(d) show the distributions of cities and users releasing Fukushima-related information, respectively. (e) the distribution of locations with respect to the number of retweets, which reflects the spatial characteristics of interesting of people, i.e., an event will arouse various attentions of people in different regions. (f-h) The daily actives of people in different time zones. Results are derived from the total data from March 9 to 31, 2011, which are normalized to 0−1. Statistical results show the consistency of sampled data in the same time zone. Time is labeled with the timestamp in UTC. Because the effect of UTC-offset (i.e., time zone), the daily active zones are different.

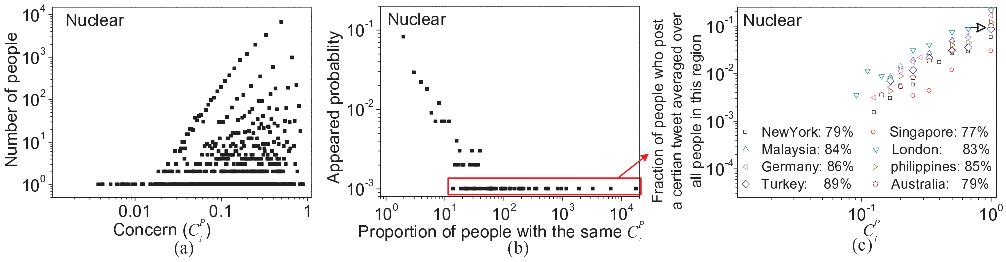

Figure G. The distribution of (a) $C^p_{nuclear}$ of people about ‘nuclear’. (b) The heterogeneous characteristics of people about ‘nuclear’. People can be divided into different groups based on the values of $C^p_{nuclear}$. (c) The relationship between the fraction of sampled people who have posted tweets containing ‘nuclear’ relative to all people in this region, and their values of $C^p_{nuclear}$ where y=1. The number in (c) stands for the percentage of statistical sampling (i.e., y=1) relative to the total in this region.

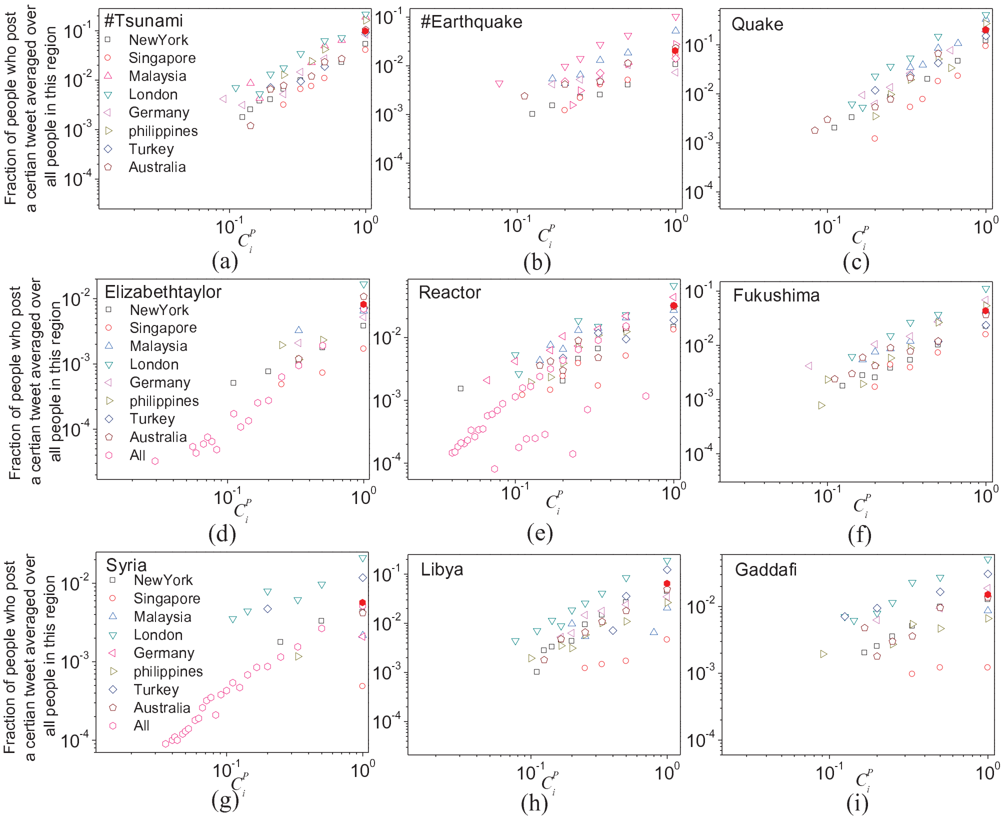

Figure H. The regional activeness during different events. People can be clustered into different groups based on the values of C_i^p, which justify the heterogeneous assumption adopted in the meat-population models for collective behaviors. A nonlinear relationship, which is not related to the type of events and regions of people, is found to characterize the relation between the proportion of people and their concerns about events as a whole. There exists an internal consistency of the distribution of concerns about events among different regions.

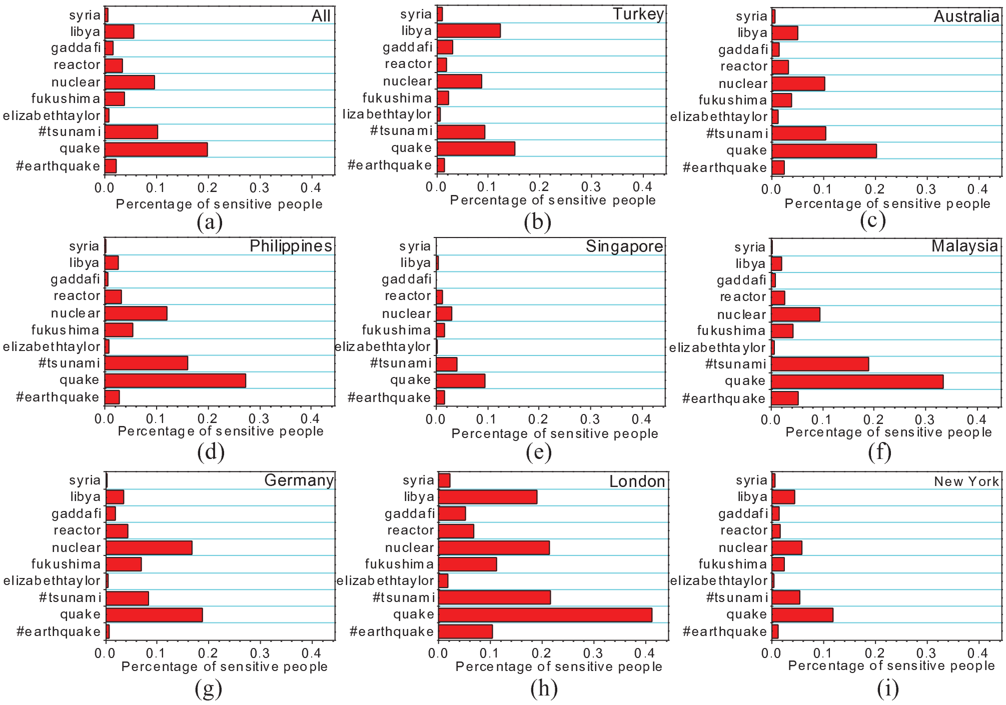

Figure I. The percentage of sensitive people for an event (i.e., $C_i^p=1$) in the same region, which reflects the heterogeneous characteristics of human concerns in same regions for different events.

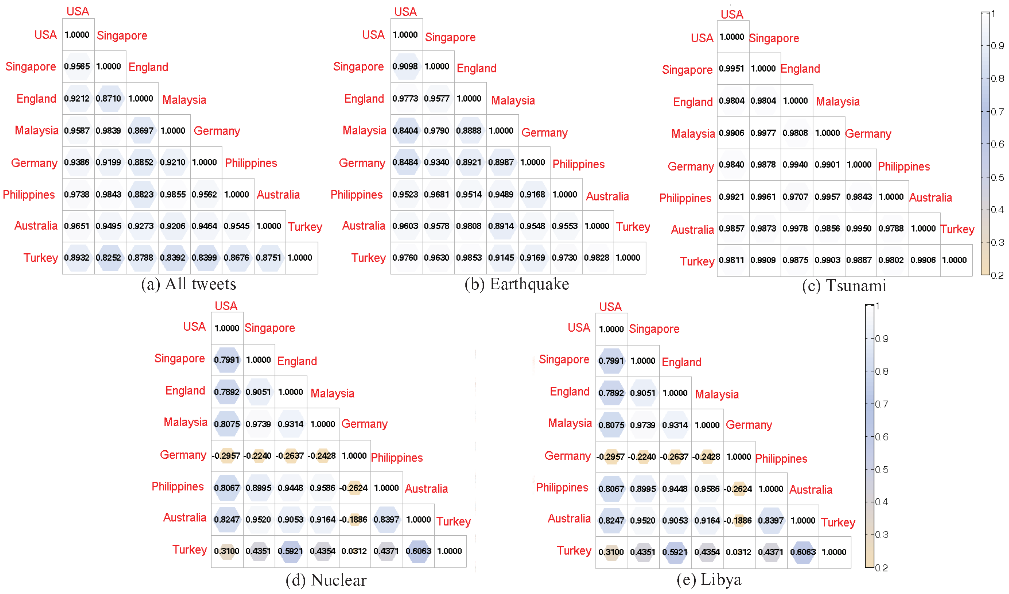

Figure J. The correlation among information spreading in different regions. There exist a heterogeneous regional responses to events based on the types of events and regional profiles.

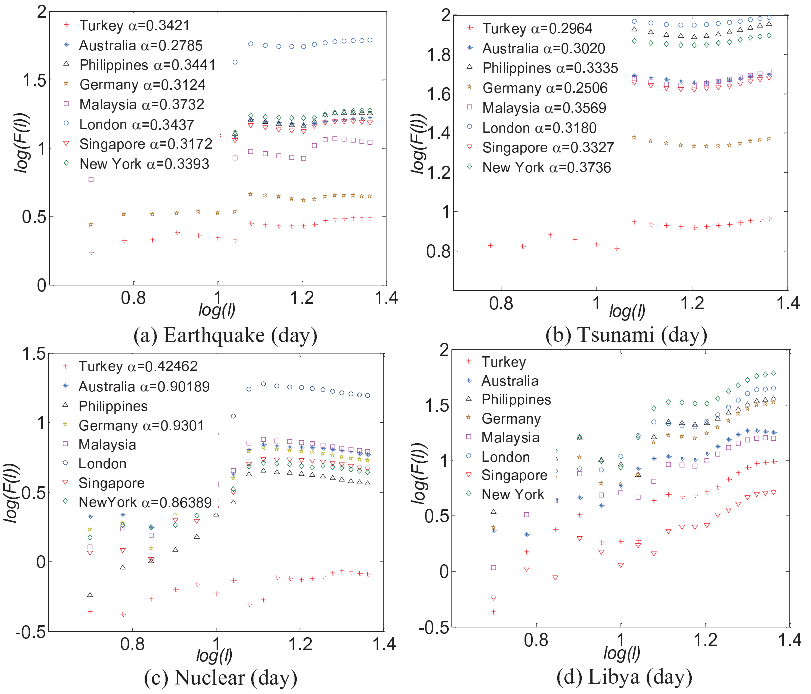

Figure K. The results of collective time series measured by DFA in a log-log plot. The symbols represent the different regional responses to the same event. The long-range correlation of human collective online behaviors is changed with time granularity. For example, the time series present the long-range correlation by the hour regardless of regional profiles and the types of events as shown in Fig 7. Yet the time series measured by the day, as shown in this figure, may not be correlated based on the type of events.

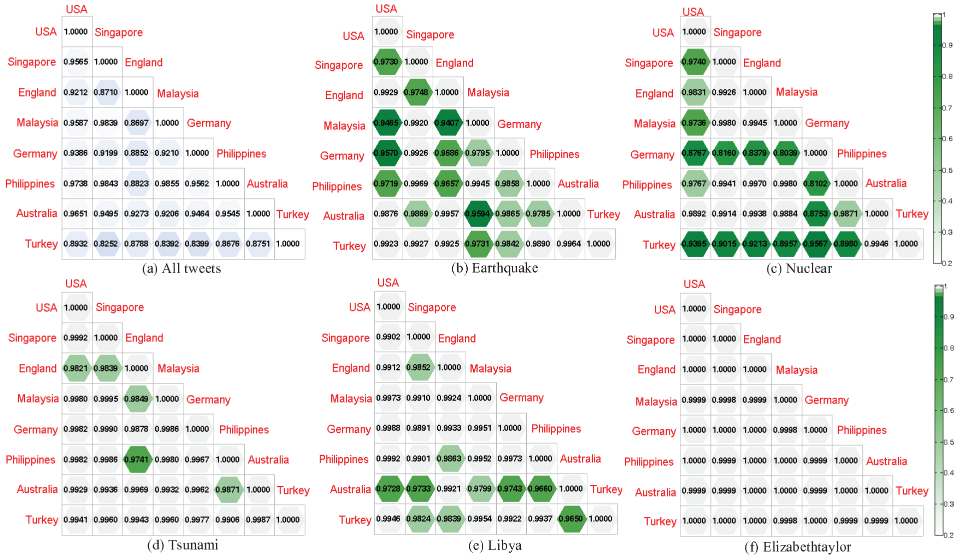

Figure L. The internal consistency of regional evolution of collective behaviors during an event. There exists a strong correlation among regions during most of events (r>0.8).
